# Prevalence of and factors associated with likely obstructive sleep apnea in individuals with airflow limitation

**DOI:** 10.3389/fmed.2024.1343372

**Published:** 2024-07-09

**Authors:** Sang Hyuk Kim, Jae Kyeom Sim, Jee Yea Choi, Ji-Yong Moon, Hyun Lee, Kyung Hoon Min

**Affiliations:** ^1^Division of Pulmonary, Allergy, and Critical Care Medicine, Department of Internal Medicine, Dongguk University Gyeongju Hospital, Dongguk University College of Medicine, Gyeongju, Republic of Korea; ^2^Division of Pulmonary, Allergy, and Critical Care Medicine, Department of Internal Medicine, Korea University Guro Hospital, Korea University College of Medicine, Seoul, Republic of Korea; ^3^Department of Internal Medicine, Hanyang University Guri Hospital, Hanyang University College of Medicine, Seoul, Republic of Korea; ^4^Division of Pulmonary Medicine and Allergy, Department of Internal Medicine, Hanyang Medical Center, Hanyang University College of Medicine, Seoul, Republic of Korea

**Keywords:** sleep apnea, obstructive, pulmonary disease, chronic obstructive, epidemiology, prevalence, airflow obstruction

## Abstract

**Introduction:**

Obstructive sleep apnea (OSA) is frequently associated with airflow limitation (AFL). However, information on the prevalence of and factors associated with likely OSA in individuals with AFL in Korea is limited.

**Methods:**

Data from the 2019 Korea National Health and Nutrition Examination Survey (KNHANES) were used, and 3,280 individuals (2,826 individuals without AFL and 454 individuals with AFL) were included. AFL was defined as forced expiratory volume in 1 s (FEV_1_)/forced vital capacity (FVC) < 0.7. A score ≥ 5 on the STOP-BANG questionnaire was used to identify individuals with likely OSA. The prevalence of likely OSA was compared between individuals with and without AFL. In addition, factors associated with likely OSA in individuals with AFL were evaluated using multivariable logistic regression analysis.

**Results:**

Of 3,280 individuals, 13.8% had an AFL. The prevalence of likely OSA was significantly higher in individuals with AFL than in individuals without AFL (9.2% vs. 5.0%, *p* = 0.014). Among 454 individuals with AFL, obesity (adjusted odds ratio [aOR] = 14.78, 95% confidence interval [CI] = 4.20–52.02) was most strongly associated with likely OSA, followed by heavy alcohol consumption (aOR = 4.93, 95% CI = 1.91–12.70), hypertension (aOR = 4.92, 95% CI = 1.57–15.46), overweight (aOR = 4.71, 95% CI = 1.76–12.64), college graduate (aOR = 4.47, 95% CI = 1.10–18.22), and history of pulmonary tuberculosis (aOR = 3.40, 95% CI = 1.06–10.96).

**Conclusion:**

In Korea, approximately 1 in 10 individuals with AFL had likely OSA. Overweight and obesity, heavy alcohol consumption, high educational level, hypertension, and history of pulmonary tuberculosis were associated with likely OSA in individuals with AFL.

## Introduction

Obstructive sleep apnea (OSA) is a common sleep-related breathing disorder characterized by upper airway obstruction during sleep, leading to fragmented sleep and daytime sleepiness (1). OSA often coexists with respiratory diseases, such as bronchial asthma and chronic obstructive pulmonary disease (COPD), characterized by airflow limitation (AFL) (2, 3). In previous studies, up to 22% of patients with OSA had comorbid AFL (4).

When OSA and obstructive airway diseases coexist, sleep quality worsens, and quality of life decreases compared with either condition alone (5, 6). Patients with OSA and obstructive airway diseases also have more frequent cardiovascular diseases (7) and a higher risk of pulmonary hypertension (8), which may lead to a higher risk of hospitalization and mortality (9). Furthermore, OSA and obstructive airway diseases potentially form a vicious cycle. OSA triggers lower airway inflammation, while reduced lung volume from these airway diseases worsens OSA severity (10). Accordingly, to improve treatment outcomes, it is important to recognize the real-world prevalence of and factors associated with OSA in individuals with obstructive airway diseases. However, information regarding this subject in Korea is limited. Although this issue was evaluated in a previous study, they used regional cohort data (11), necessitating analysis using national representative information.

This study aimed to investigate the prevalence of and factors associated with likely OSA in individuals with AFL. For the current study, we used Korea National Health and Nutrition Survey (KNHANES) data, which includes spirometry results and questionnaires assessing likely OSA.

## Materials and methods

### Study population

We used the 2019 KNHANES, a cross-sectional nationwide survey that assesses the health and nutritional status of non-institutionalized Korean citizens. The KNHANES is designed, managed, and monitored by the Korea Disease Control and Prevention Agency (KDCA). The study participants were chosen based on a stratified multistage probability cluster sampling method. In brief, the KNHANES has collected data on demographics and personal habits, including age, sex, height, weight, and self-reported smoking history. It also includes spirometry results in participants aged ≥40 years. More detailed information regarding the KNHANES can be found in previous studies (12–16).

**Figure 1:**
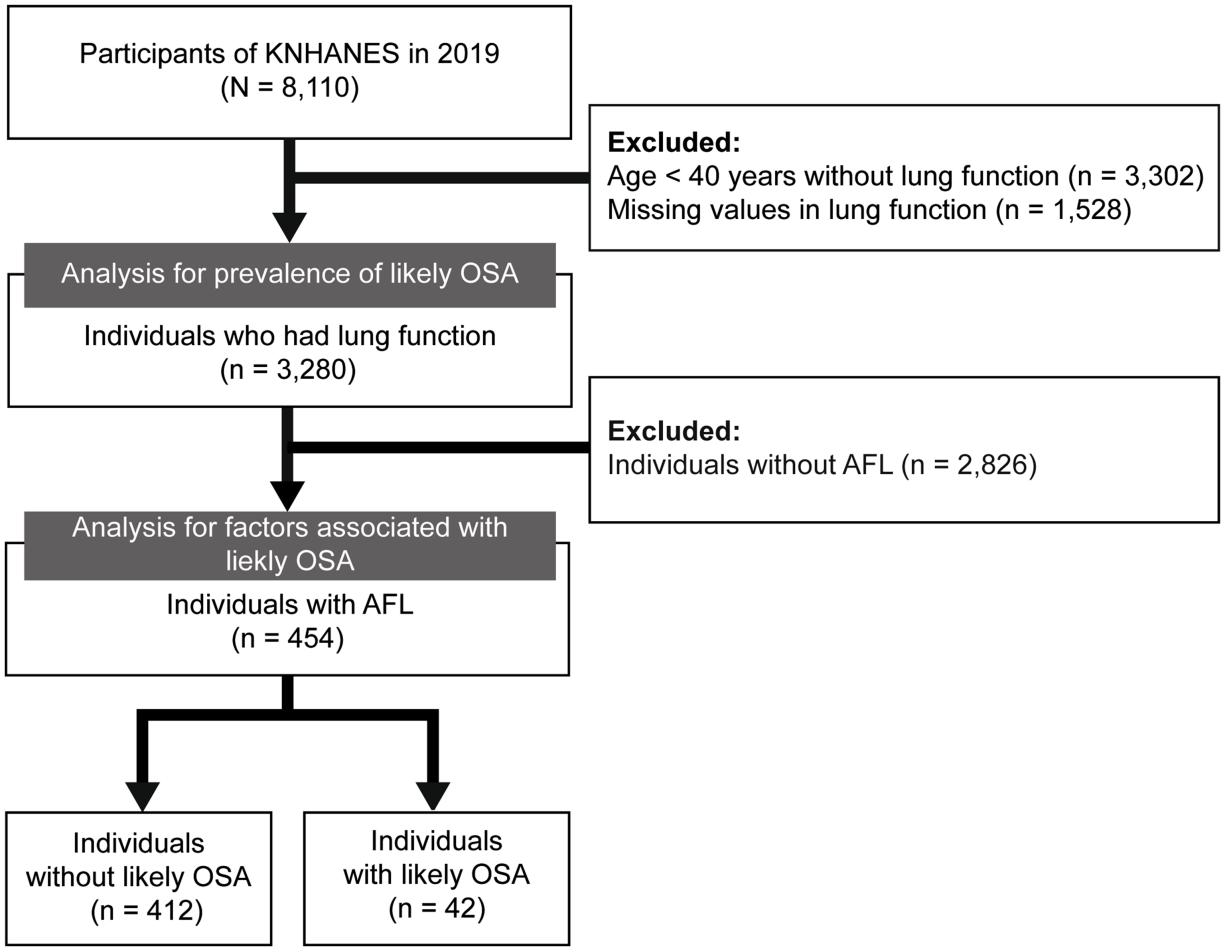
Flow chart of the study population. KNHANES, Korea National Health and Nutrition Examination Survey; OSA, obstructive sleep apnea; AFL, airflow limitation.

A total of 8,110 individuals were included in the 2019 KNHANES. After excluding 3,302 individuals aged ≤40 who did not perform spirometry, and 1,528 individuals with missing values in lung function measurements, 3,280 individuals were included in the prevalence analysis. In addition, 2,826 individuals without AFL were further excluded, leaving 454 individuals in the final analytic cohort for assessing factors associated with likely OSA in individuals with AFL (Figure 1).

### Definitions: AFL and likely OSA

Spirometry was assessed by well-trained technicians using a spirometer (Vyntus Spiro; Care Fusion, San Diego, CA, United States; or dry rolling seal spirometer Model 2,130; Sensormedics Corporation, Yorba Linda, CA, United States) following the American Thoracic Society (ATS)/European Respiratory Society (ERS) recommendations (17). Since KNHANES provides only pre-bronchodilator values, AFL was defined as the pre-bronchodilator value of forced expiratory volume in 1 s (FEV_1_)/forced vital capacity (FVC) < 0.7.

Likely OSA, suggesting a high risk for OSA, was assessed using a questionnaire on snoring, tiredness, observed apnea, blood pressure, body mass index (BMI), age, neck circumference, and gender (STOP-BANG). Likely OSA was defined as a STOP-BANG score ≥ 5, which is generally considered high risk. More detailed information on the STOP-BANG questionnaire was described in the previous studies (18–20).

### Covariates

BMI was calculated as weight in kilograms divided by height in meters squared (kg/m^2^). Past smokers were defined as individuals with a history of smoking ≥5 cigarettes in their lifetime but did not currently smoke. Heavy drinking was defined as consuming >30 g/day of alcohol. Individuals who were single, separated, divorced, or widowed were considered unmarried. Income was categorized as low, intermediate, or high based on the tertiles of monthly personal income. Physical activity was determined using the Global Physical Activity Questionnaire based on intensity, frequency, and time. Depressive mood was defined as sadness for at least 2 weeks. Hypertension was defined as systolic blood pressure ≥ 140 mmHg, diastolic blood pressure ≥ 90 mmHg, use of antihypertensive drugs, or previous physician diagnosis. Diabetes mellitus was defined as fasting glucose level ≥ 126 mg/dL, use of insulin or antidiabetic drugs, or previous physician diagnosis. Dyslipidemia was defined as low-density lipoprotein cholesterol >130 mg/dL, high-density lipoprotein cholesterol <40 mg/dL, use of antidyslipidemic drugs, or previous physician diagnosis of dyslipidemia. Cardiovascular disease was defined as physician diagnosis of myocardial infarction or angina pectoralis. Educational level, respiratory symptoms (cough and sputum), sleep duration, stress, and other comorbidities (asthma, history of pulmonary tuberculosis) were assessed by self-reported questionnaires. Detailed definitions of comorbidities were described in our previous studies (21–28).

### Statistical analysis

Data are expressed as weighted percentages with standard error (SE). The *p*-values for evaluating intergroup differences were calculated using Pearson’s chi-square test. We compared the prevalence of likely OSA in individuals with (*n* = 454) and without AFL (*n* = 2,826). The calculated power using post-hoc power analysis was 92.8% before adjusting for survey weights (29).

Multivariable logistic regression analysis was used to explore the factors associated with likely OSA in individuals with AFL. Two models were developed for multivariable analysis. Model 1 (excluding variables used to define OSA) included sociodemographic variables (educational level, income), habitual behaviors (smoking, heavy alcohol consumption), clinically significant variables (physical activity, respiratory symptoms, sleep duration), percentage of predicted FEV1 (FEV1%pred), and comorbidities (diabetes mellitus, dyslipidemia, cardiovascular disease, asthma, history of pulmonary tuberculosis). Model 2 included the variables from Model 1 and additional variables used to define OSA (age, BMI, and hypertension). Sex was not included due to the limited number of OSA cases in women. Weighting adjustments were implemented in all analyses and statistical significance was determined based on a two-sided *p*-value <0.05. All analyses were conducted in R version 4.3.1 (R Core Team 2023; R Foundation for Statistical Computing in Vienna, Austria).

## Results

### Prevalence of likely OSA in individuals with AFL

**Figure 2:**
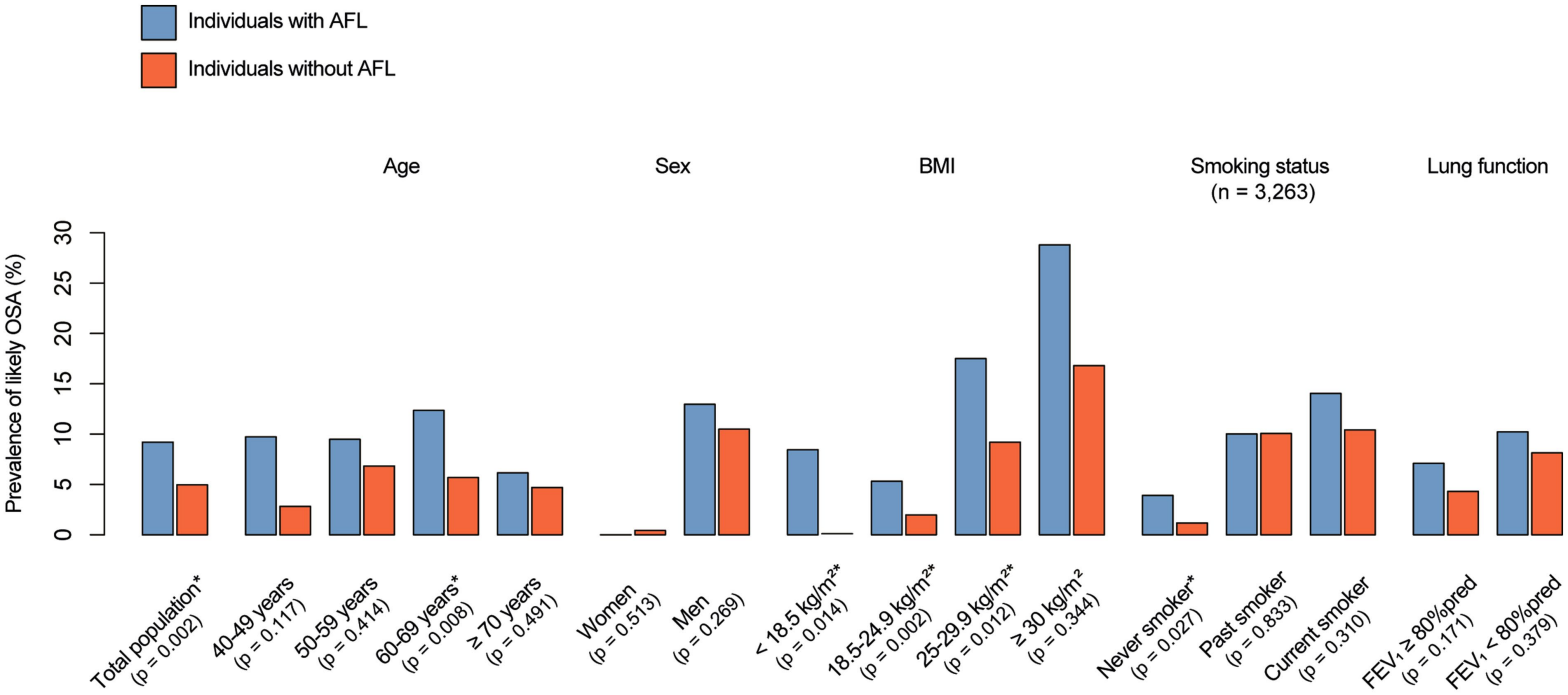
Prevalence of likely OSA and AFL. The *p*-value was calculated using a weight-adjusted chi-square test, with *indicating *p* < 0.05. OSA, obstructive sleep apnea; AFL, airflow limitation; BMI, body mass index.

As shown in Figure 2, individuals with AFL were more likely to have likely OSA than were individuals without AFL (9.2 vs. 5.0%, *p* = 0.002). In subgroup analysis, the prevalence of likely OSA differed between the individuals with and without AFL, showing a pronounced gap in prevalence of likely OSA for younger individuals (9.7 vs. 2.8% for the 40–49 age group, *p* = 0.117), underweight individuals (8.5 vs. 0%, *p* = 0.014), and never-smokers (3.9 vs. 1.2%, *p* = 0.027). Detailed information on the prevalence of likely OSA based on subgroup (age, sex, BMI category, smoking status, and lung function) is shown in Figure 2.

### Baseline characteristics

Table 1 shows the baseline characteristics of the study population. Among individuals with AFL, three-fourths were > 60 years of age, and two-thirds were male. Individuals with likely OSA were more likely to be male than those without likely OSA (100 vs. 68.0%, *p* < 0.001); however, age was similar between individuals with or without likely OSA. Individuals with likely OSA were taller (170.6 vs. 164.4 cm, *p* < 0.001), heavier (75.8 vs. 63.5 kg, *p* < 0.001), more likely to be overweight or obese (58.5 vs. 26.2%, *p* < 0.001), had a thicker neck circumference (39.7 vs. 35.8 cm, *p* < 0.001), had higher rate of a history of smoking (86.1 vs. 65.5%, *p* < 0.001), consumed more alcohol (heavy drinker, 40.1 vs. 14.5%, *p* < 0.001), and were more likely to be married (94.8 vs. 76.9%, *p* < 0.001) than individuals without likely OSA. There were no significant intergroup differences in socioeconomic status (income and education), physical activity, respiratory symptoms, sleep duration, and mental health (*p* > 0.05 for all variables).

**Table 1:** Baseline characteristics of the AFL study population.

	Total population (*n* = 454)	Without likely OSA (*n* = 412)	With likely OSA (*n* = 42)	*p*-value
Age, years				0.458
40–49	7.1 (1.5)	7.1 (1.6)	7.5 (5.7)	
50–59	20.9 (2.7)	20.8 (2.9)	21.6 (7.7)	
60–69	33.6 (2.6)	32.4 (2.7)	45.2 (7.5)	
≥ 70	38.4 (2.9)	39.7 (3.1)	25.7 (8.8)	
Sex				< 0.001
Female	29.1 (2.5)	32.0 (2.6)	0 (0)	
Male	70.9 (2.5)	68.0 (2.6)	100 (0)	
Height, cm	164.9 (0.5)	164.4 (0.5)	170.6 (1.2)	< 0.001
Weight, kg	64.6 (0.6)	63.5 (0.5)	75.8 (1.9)	< 0.001
BMI, kg/m^2^				< 0.001
< 18.5 (underweight)	1.9 (0.7)	1.9 (0.8)	1.7 (1.8)	
18.5–24.9 (normal weight)	68.9 (2.5)	71.9 (2.5)	39.9 (8.5)	
25–29.9 (overweight)	26.9 (2.5)	24.4 (2.5)	51.1 (8.0)	
≥ 30 (obese)	2.4 (0.8)	1.8 (0.8)	7.4 (3.9)	
Neck circumference, cm (*n* = 453)	36.2 (0.2)	35.8 (0.2)	39.7 (0.4)	< 0.001
Smoking status (*n* = 453)				0.022
Non-smoker	32.6 (2.5)	34.5 (2.6)	13.8 (6.4)	
Past smoker	37.6 (2.5)	37.3 (2.7)	40.8 (7.1)	
Current smoker	29.8 (2.7)	28.2 (2.9)	45.3 (6.3)	
Smoking amount, pack years				< 0.001
< 10	41.8 (2.7)	44.2 (2.8)	17.6 (7.2)	
10–19	12.4 (1.7)	12.4 (1.8)	12.2 (5.4)	
≥ 20	45.8 (2.9)	43.4 (2.9)	70.2 (8.6)	
Heavy alcohol consumption (*n* = 453)	16.8 (2.1)	14.5 (2.1)	40.1 (9.1)	< 0.001
Marital status				0.018
Unmarried	21.5 (2.2)	23.1 (2.4)	5.2 (3.9)	
Married	78.5 (2.2)	76.9 (2.4)	94.8 (3.9)	
Income (*n* = 451)				0.834
Low	24.1 (2.0)	23.7 (2.1)	28.0 (6.0)	
Intermediate	48.2 (2.6)	48.4 (2.7)	45.6 (5.4)	
High	27.7 (2.6)	27.9 (2.6)	26.4 (6.5)	
Educational level (*n* = 425)				0.235
Elementary school graduate	28.5 (2.7)	29.3 (3.0)	20.7 (6.3)	
Middle/high school graduate	50.1 (3.0)	50.5 (3.1)	46.4 (10.5)	
College graduate	21.5 (2.7)	20.2 (3.0)	32.9 (8.4)	
Physical activity, METs-min/week (*n* = 425)				0.620
< 500	55.2 (2.7)	55.8 (2.9)	49.8 (8.5)	
500–999	16.7 (1.9)	16.9 (1.9)	14.7 (6.5)	
≥ 1,000	28.1 (2.6)	27.3 (2.8)	35.5 (7.9)	
Respiratory symptoms, any	10.9 (1.5)	10.5 (1.61)	15.2 (5.0)	0.378
Cough	7.2 (1.3)	7.1 (1.4)	8.2 (4.5)	0.808
Sputum	8.2 (1.1)	7.80 (1.2)	12.0 (3.9)	0.330
Sleep duration, hours (*n* = 452)				0.833
< 8	71.7 (2.4)	71.6 (2.5)	73.1 (7.4)	
≥ 8	28.3 (2.4)	28.4 (2.5)	26.9 (7.4)	
Mental health (*n* = 453)				
Stress	18.8 (2.4)	18.5 (2.5)	22.1 (7.7)	0.622
Depressive mood	9.0 (1.3)	9.4 (1.4)	4.9 (3.0)	0.299
Lung function				
FVC, L	3.4 (0.1)	3.4 (0.1)	3.8 (0.1)	0.003
FVC, %pred	84.8 (0.8)	85.1 (0.9)	81.8 (2.0)	0.176
FEV_1_, L	2.2 (0.0)	2.1 (0.0)	2.4 (0.1)	0.014
FEV_1_, %pred	74.1 (0.8)	74.3 (0.9)	71.9 (1.8)	0.300
FEV_1_/FVC, %	63.6 (0.3)	63.6 (0.3)	63.3 (1.4)	0.830
Comorbidities				
Hypertension	50.7 (2.8)	48.0 (3.1)	78.1 (4.9)	0.001
Diabetes mellitus	18.9 (1.9)	18.1 (1.9)	26.1 (7.8)	0.245
Dyslipidemia	46.9 (2.7)	45.6 (2.9)	60.1 (8.5)	0.132
Cardiovascular disease (*n* = 426)	3.6 (1.0)	3.0 (1.0)	9.6 (5.6)	0.063
Asthma	5.3 (1.1)	5.1 (1.1)	7.6 (4.3)	0.529
History of pulmonary tuberculosis	8.3 (1.4)	7.6 (1.4)	15.0 (6.8)	0.167

AFL, airflow limitation; MET, metabolic equivalent of task; FVC, forced vital capacity; FEV_1_, forced expiratory volume in 1 s; BMI, body mass index; %pred, percentage of predicted.

Regarding lung function, individuals with likely OSA had higher FVC (3.8 vs. 3.4 L, *p* = 0.003) and FEV_1_ (2.4 vs. 2.1 L, *p* = 0.014) than individuals without likely OSA; however, FVC %pred, FEV_1_%pred, and FEV_1_/FVC ratio did not differ between individuals with and without likely OSA (*p* > 0.05 for all variables). Among comorbidities, individuals with likely OSA were more likely to have hypertension (78.1% vs. 48.0%, *p* = 0.001). However, there were no differences in other comorbidities (*p* > 0.05 for all variables).

### Factors associated with likely OSA in individuals with AFL

Table 2 shows the results of multivariable logistic regression analysis for factors associated with likely OSA among individuals with AFL. In model 1, only heavy alcohol consumption was associated with likely OSA (adjusted odds ratio [aOR] = 3.50, 95% confidence interval [CI] = 1.44–8.49). In Model 2, obesity was the factor most strongly associated with likely OSA (aOR = 14.78, 95% CI = 4.20–52.02). Other factors significantly associated with likely OSA were overweight (aOR = 4.71, 95% CI = 1.76–12.64), heavy alcohol consumption (aOR = 4.93, 95% CI = 1.91–12.70), college graduate (aOR = 4.47, 95% CI = 1.10–18.22), hypertension (aOR = 4.92, 95% CI = 1.57–15.46), and history of pulmonary tuberculosis (aOR = 3.40, 95% CI = 1.06–10.96).

**Table 2:** Factors associated with likely OSA in individuals with AFL.

*n* = 454	Univariable analysis	Model 1^*^	Model 2^*^
	OR (95% CI)	Adjusted OR (95%CI)	Adjusted OR (95%CI)
Age, years			
40–49	Ref.	–	Ref.
50–59	0.97 (0.15–6.26)	–	1.25 (0.14–11.16)
60–69	1.31 (0.26–6.64)	–	2.74 (0.40–18.67)
≥ 70	0.61 (0.10–3.83)	–	2.04 (0.21–19.48)
BMI			
Underweight	1.64 (0.18–15.00)	–	4.08 (0.37–44.49)
Normal weight	Ref.	–	Ref.
Overweight	**3.77 (1.86–7.62)**	–	**4.71 (1.76–12.64)**
Obese	**7.20 (1.70–30.49)**	–	**14.78 (4.20–52.02)**
Smoking status (*n* = 453)			
Non-smoker	Ref.	Ref.	Ref.
Past smoker	2.73 (0.90–8.32)	1.84 (0.59–5.72)	1.76 (0.49–6.31)
Current smoker	4.01 (1.42–11.31)	2.42 (0.73–8.09)	3.26 (0.87–12.22)
Heavy alcohol consumption (*n* = 453)			
No	Ref.	Ref.	Ref.
Yes	**3.96 (1.90–8.24)**	**3.50 (1.44–8.49)**	**4.93 (1.91–12.70)**
Income (*n* = 451)			
Low	1.25 (0.52–3.01)	1.55 (0.53–4.52)	1.31 (0.35–4.92)
Intermediate	1.00 (0.45–2.20)	1.29 (0.51–3.21)	1.46 (0.49–4.30)
High	Ref.	Ref.	Ref.
Educational level (*n* = 425)			
Elementary school graduate	Ref.	Ref.	Ref.
Middle/high school graduate	1.30 (0.52–3.26)	0.98 (0.39–2.46)	1.54 (0.54–4.40)
College graduate	2.30 (0.86–6.12)	2.11 (0.63–7.06)	**4.47 (1.10–18.22)**
Physical activity, METs-min/week (*n* = 425)			
< 500	Ref.	Ref.	Ref.
500–999	0.97 (0.32–2.99)	0.93 (0.29–3.01)	1.17 (0.38–3.57)
≥ 1,000	1.46 (0.65–3.28)	1.14 (0.62–7.06)	1.38 (0.51–3.71)
Respiratory symptoms, any			
No	Ref.	Ref.	Ref.
Yes	1.53 (0.59–3.94)	1.36 (0.41–4.56)	2.45 (0.66–9.12)
Sleep duration, hours (*n* = 452)			
< 8	Ref.	Ref.	Ref.
≥ 8	0.93 (0.45–1.90)	1.56 (0.72–3.38)	1.60 (0.66–3.89)
Lung function			
FEV_1_, per 10%pred decrease	1.10 (0.92–1.33)	1.08 (0.88–1.32)	1.03 (0.82–1.28)
Comorbidities			
Hypertension	**3.87 (1.75–8.57)**	**-**	**4.92 (1.57–15.46)**
Diabetes mellitus	1.59 (0.73–3.49)	1.51 (0.56–4.04)	1.12 (0.42–3.01)
Dyslipidemia	1.80 (0.84–3.87)	1.80 (0.74–4.34)	0.96 (0.34–2.73)
Cardiovascular disease (n = 426)	3.43 (0.88–13.43)	4.35 (0.99–19.21)	4.29 (0.68–27.06)
Asthma	1.53 (0.40–5.82)	0.97 (0.19–4.84)	1.40 (0.30–6.56)
History of pulmonary tuberculosis	2.14 (0.72–6.39)	2.48 (0.86–7.16)	**3.40 (1.06–10.96)**

*420 individuals were included in the multivariable analysis. Bold texts indicate statistically significant results. OR, odds ratio; CI, confidence interval; MET, metabolic equivalent of task; FEV_1_, forced expiratory volume in 1 s; BMI, body mass index.

## Discussion

This nationwide, representative, cross-sectional study evaluated the prevalence of likely OSA and factors associated with likely OSA in individuals with AFL. Our study showed that approximately 1 in 10 individuals with AFL had likely OSA, which was significantly higher than in individuals without AFL. In addition, the prevalence gap of likely OSA between individuals with and without AFL was greater in younger and underweight individuals and never-smokers. Overweight or obesity, heavy alcohol consumption, high education, hypertension, and history of pulmonary tuberculosis were associated with increased odds of having likely OSA in individuals with AFL.

The prevalence of OSA in individuals with COPD varies depending on the definition of OSA and COPD. OSA is a common comorbidity in individuals with COPD, affecting approximately 60% of patients (30–32). In the present study, the prevalence of likely OSA in individuals with AFL was 9.2%, which was significantly higher than in individuals without AFL (5.0%). However, this rate was relatively lower than previously reported rates of comorbid OSA in individuals with COPD.

There are some possible explanations for this finding. First, not all individuals with AFL in this study can be classified as those with COPD based on traditional definition of COPD (21). Several other diseases and conditions can cause AFL. However, according to the recently revised GOLD definition for COPD, most individuals with AFL would be considered to have COPD (33). Moreover, there is an emerging concept advocating for more proactive strategies to diagnose COPD early, including the identification of pre-COPD. In this context, it seems acceptable that most individuals with AFL fall within the category of COPD. Second, the participants in our study were enrolled in a nationwide survey. Thus, a considerable proportion was more likely to be clinically stable, less symptomatic, and have better lung function than individuals from COPD cohorts (12, 34). The severity of COPD is associated with the risk of comorbid OSA (35). However, the strength of the present study is the use of a nationwide representative sample, and it was the first to outline the scheme on the overall burden of OSA in Koreans with AFL.

An important finding of our study is that individuals with AFL, particularly younger individuals, underweight individuals, and never-smokers, had a higher prevalence of likely OSA than individuals without AFL. This suggests that populations not typically at risk for OSA may have an increased risk if they have AFL. This observation has important implications, particularly for certain patients (i.e., pre-COPD, COPD in young people, individuals with sarcopenia, and never-smokers) who have recently attracted attention due to their potentially distinctive characteristics compared with the traditional COPD phenotype (36–38). Although the exact mechanisms are unclear, increasing evidence indicates that pathogenesis and clinical manifestations of COPD may differ in these patients compared to patients with classic smoking COPD (39). Our study suggests that comorbid OSA may be an additional potential explanation for underlying pathophysiology. Therefore, future studies on the role of OSA in these entities, such as pre-COPD, COPD in young people, sarcopenia, and never-smokers, are warranted.

We identified several factors associated with an increased risk of likely OSA in individuals with AFL. It is uncertain whether including variables used to define likely OSA, such as age, BMI, and hypertension, is necessary for the multivariable analysis. However, in the fully adjusted model that includes these variables, we identified additional factors, indicating the need to adjust these variables. Results from the fully adjusted model are similar to previous findings, showing that individuals who are overweight or obese, consume a large amount of alcohol, or have hypertension are more likely to have OSA (40–42). In addition, a history of pulmonary tuberculosis, which often results in respiratory sequelae, was associated with an increased risk of likely OSA in individuals with AFL. In alignment with the results from a previous study, lung function, as well as the quality of sleep, were worse in patients with tuberculosis compared with their non-tuberculosis family members (43). However, in contrast to previous studies, higher educational level was unexpectedly associated with an increased risk of likely OSA in the present study (44, 45), warranting further research.

It is gradually recognized that the coexistence of OSA in COPD has important clinical relevance (46). This recognition led to the emergence of the concept of COPD–OSA overlap syndrome, and several studies reported that this disease entity has been linked with a poor prognosis (7, 47). Reduced lung volume is associated with increased OSA severity, potentially due to increased upper airway collapsibility (1). Supporting this notion, patients with OSA who had reduced lung function exhibited a higher risk of death. Nevertheless, both diseases are frequently underdiagnosed (48, 49) despite available treatment options to reduce the burden of both COPD and OSA (50, 51). Therefore, it is imperative to consider strategies for early detection and effective management of both conditions.

There are some limitations in our study. First, we used the STOP-BANG questionnaire to define likely OSA rather than sleep polysomnography, which is the gold standard for diagnosing OSA (1). In addition, there has still been debate about the clinical utility of various questionnaires for screening OSA (52, 53). Nevertheless, the STOP-BANG questionnaire has been validated in several previous studies as a useful tool for identifying OSA (18, 54). Also, we expressed this condition as likely OSA, suggesting a high risk of OSA rather than a diagnosis of OSA. Second, as mentioned above, pre-bronchodilator lung function measurements allowed us to include individuals with AFL, which can mostly be classified as COPD. However, COPD is a heterogeneous condition, and our results may not represent specific COPD phenotypes or etiologies. Therefore, our findings should be generalized cautiously and interpreted from an epidemiological perspective. Third, the prevalence of OSA may be overestimated because individuals with COPD frequently exhibit symptoms similar to those found in OSA, particularly when OSA is assessed using a questionnaire. Fourth, we used only data from 1 year, which limited the number of study participants. Because KNHANES is an ongoing study, research using multiannual data will be possible in future studies. Fifth, we could not provide information when the lung function was measured. Since individuals with OSA had an evening-to-morning variability in lung function, our study results might have been influenced by this variability (55). Sixth, our results are based on the Korean population, and caution is needed when interpreting and applying the findings to populations in other countries.

In conclusion, approximately 1 in 10 individuals with AFL in Korea had likely OSA. Overweight and obesity, heavy alcohol consumption, high educational level, hypertension, and history of tuberculosis were associated with likely OSA in individuals with AFL.

## Data availability statement

Publicly available datasets were analyzed in this study. This data can be found at: https://knhanes.kdca.go.kr/knhanes/main.do.

## Ethics statement

The studies involving humans were approved by Institutional Review Board (IRB) of the Korea Disease Control and Prevention Agency (KDCPA) approved the protocol (IRB No. 2018-01-03-C-A). The studies were conducted in accordance with the local legislation and institutional requirements. The participants provided their written informed consent to participate in this study.

## Author contributions

SHK: Data curation, Formal analysis, Visualization, Writing – original draft. JKS: Writing – original draft. JYC: Writing – original draft. J-YM: Writing – review & editing. HL: Supervision, Validation, Writing – review & editing. KHM: Supervision, Validation, Writing – review & editing.
